# Contempt of Court

**Published:** 1888-02

**Authors:** 


### CONTEMPT OF COURT.

The *St. Louis Medical Journal* says: "Of all the curious reading that we have enjoyed in some time, we think that afforded by a communication from Dr. F. E. Stewart to the current number of the *Druggists' Circular* certainly caps the climax. It affords a splendid illustration of the wisdom of the adage which advises the shoemaker to stick to his last. Whenever a physician strays from his own profession into the intricacies of the law, and especially of the patent laws of this country, his feet are in dangerous and slippery ground, no matter where his head or heart may be. In the present paper, Dr. Stewart attacks the recent decision of the United States District Court in the matter of the suit of Battle & Co. against the Grosses (Daniel W. and Edward Z.) for infringement of their copyright of Bromidia. He declares that the decision is not final or binding, and advises the Grosses and druggists generally not to pay any attention to it. Dr. Stewart thus puts himself in contempt of the United States Courts and advises others to place themselves in the same foolish and dangerous predicament. The queer part of the matter, however, is that every reason which he advances against the validity and justice of the decision is the strongest possible argument in its favor, and the reader must be obtuse ndeed not to see that it is so. This view of it was evidently taken by the editor of the *Circular*, who says: "While giving Dr. Stewart's argument publicity on account of its novelty, we think it proper to remind pharmacists that they are bound by the decision so long as it is allowed to stand"—which advice is good, sound sense, like pretty much everything that eminates from the editor of the journal quoted."

